# 

**Published:** 1897-12

**Authors:** 


### CONTENTS.

ORIGINAL COMMUNICATIONS. PAGE

Regulating without Extraction *versus* Extraction for Regulating; Some Typical Comparative Results. By WM. SLOCUM DAVENPORT, D.D.S., Paris, France . . 777
Dr. G. V. Black's Conclusions reviewed. By CHARLES S. TOMES, F.R.S., London, England . . . . . . 781
Dr. G. V. Black's Conclusions reviewed. By DR. R. R. ANDREWS, Boston, Mass. 785
Dr. G. V. Black's Conclusions reviewed. By DR. S. B. PALMER, Syracuse, N. Y. 789
Dr. G. V. Black's and Dr. J. Leon Williams's Conclusions reviewed. By JAMES TRUMAN, D.D.S. . . . . . 796
Dr. G. V. Black's Conclusions reviewed. By GEORGE A. MAXFIELD, D.D.S., Holyoke, Mass. . . . . . 807
Dr. G. V. Black's Conclusions reviewed. By DR. E. A. BOGUE, New York . . . 812
Dr. G. V. Black's Conclusions reviewed. By DR. B. HOLLY SMITH, Baltimore, Md. 814

ABSTRACTS AND TRANSLATIONS.

A Few Considerations on the Leptothrix Buccalis. By T. CHOQUET, D.E.D.P., Paris . . . . . . 816

REPORTS OF SOCIETY MEETINGS.

American Dental Association . . . . . . 822
The New York Institute of Stomatology . . . . . . 826

EDITORIAL.

The Record of the Year . . . . . . 832
Southern Dental Association . . . . . . 835
Correction . . . . . . 835

BIBLIOGRAPHY . . . . . . 836

OBITUARY.

Dr. Thomas W. Evans . . . . . . 841
Dr. Harrison Allen . . . . . . 844

CURRENT NEWS.

Programme of the Rochester Dental Society, Sessions of 1897–98 . . . . . . 846
Northern Illinois Dental Society . . . . . . 847

SELECTIONS.

Carious Teeth as a Means of Entry into the Body for the Bacillus of Tuberculosis . 848

